# Ornithine decarboxylase as a therapeutic target for endometrial cancer

**DOI:** 10.1371/journal.pone.0189044

**Published:** 2017-12-14

**Authors:** Hong Im Kim, Chad R. Schultz, Andrea L. Buras, Elizabeth Friedman, Alyssa Fedorko, Leigh Seamon, Gadisetti V. R. Chandramouli, G. Larry Maxwell, André S. Bachmann, John I. Risinger

**Affiliations:** 1 Department of Obstetrics, Gynecology and Reproductive Biology, Michigan State University, Grand Rapids, Michigan, United States of America; 2 Department of Pediatrics and Human Development, Michigan State University, Grand Rapids, Michigan, United States of America; 3 Spectrum Health, Grand Rapids, Michigan, United States of America; 4 Genepria Consulting, Columbia, Maryland, United States of America; 5 Department of Obsteterics and Gynecology, Inova Fairfax Women’s Hospital, Falls Church, Virginia, United States of America; ENEA Centro Ricerche Casaccia, ITALY

## Abstract

Ornithine Decarboxylase (ODC) a key enzyme in polyamine biosynthesis is often overexpressed in cancers and contributes to polyamine-induced cell proliferation. We noted ubiquitous expression of *ODC1* in our published endometrial cancer gene array data and confirmed this in the cancer genome atlas (TCGA) with highest expression in non-endometrioid, high grade, and copy number high cancers, which have the worst clinical outcomes. *ODC1* expression was associated with worse overall survival and increased recurrence in three endometrial cancer gene expression datasets. Importantly, we confirmed these findings using quantitative real-time polymerase chain reaction (qRT-PCR) in a validation cohort of 60 endometrial cancers and found that endometrial cancers with elevated *ODC1* had significantly shorter recurrence-free intervals (KM log-rank p = 0.0312, Wald test p = 5.59e-05). Difluoromethylornithine (DFMO) a specific inhibitor of ODC significantly reduced cell proliferation, cell viability, and colony formation in cell line models derived from undifferentiated, endometrioid, serous, carcinosarcoma (mixed mesodermal tumor; MMT) and clear cell endometrial cancers. DFMO also significantly reduced human endometrial cancer ACI-98 tumor burden in mice compared to controls (p = 0.0023). ODC-regulated polyamines (putrescine [Put] and/or spermidine [Spd]) known activators of cell proliferation were strongly decreased in response to DFMO, in both tumor tissue ([Put] (p = 0.0006), [Spd] (p<0.0001)) and blood plasma ([Put] (p<0.0001), [Spd] (p = 0.0049)) of treated mice. Our study indicates that some endometrial cancers appear particularly sensitive to DFMO and that the polyamine pathway in endometrial cancers in general and specifically those most likely to suffer adverse clinical outcomes could be targeted for effective treatment, chemoprevention or chemoprevention of recurrence.

## Introduction

Endometrial cancer is the most common gynecological malignancy in developed countries. In the United States it is the 4^th^ most common cancer and the 6^th^ most deadly cancer in women. Unlike most other common cancers the numbers of endometrial cancers are increasing and the death rate rises annually. To this point recent American Cancer Society estimates indicate there will be 61,380 newly diagnosed uterine corpus cancers and 10,920 deaths in 2017[1]. The NCI indicates the death rate will rise again this year, a trend that has continued for over 40 straight years[2]. These increases are likely due to increased obesity, a major risk factor for endometrial cancer and to age trends of the population. Neither of these trends are expected to change in coming years with 122,000 annual new cases expected by 2030[3]. Most endometrial cancers are detected early (stage 1 uterine confined disease) and are subsequently cured by hysterectomy alone or in combination with adjuvant chemotherapies or radiation[4,5]. However, recurrent disease and advanced disease remain a significant clinical challenge with limited therapeutic options available. Patients with local recurrence (stage II) have an approximately 68% 5-year survival rate, stage III tumors 47–58% whereas those with distant recurrence and metastasis a dismal 15–17%[2,6–8].

Prediction of which endometrial cancer patients will recur has been a decade’s long obstacle for clinicians, and researchers. Long held clinical and pathologic assessments based on important tumor characteristics including tumor stage, grade, and histologic type which guide current care. While generally applied these criteria often fail to accurately predict tumor recurrence which can lead to the over and under treatment of patients. The long held belief in the field that molecular classification will enhance or replace existing clinical and pathologic determinations is still in progress and has not and may not come to fruition. Therefore, any treatments or chemopreventive agents should target a gene or pathway present in as many endometrial cancers as possible for it to be broadly effective. Furthermore, if the target is broadly expressed and importantly in those cases most likely to progress this will further enhance the desirability of the target.

Ornithine decarboxylase (ODC) is the rate-limiting enzyme in polyamine biosynthesis and involved in the production of putrescine through the decarboxylation of ornithine[9,10]. ODC is often overexpressed in cancerous tissues and contributes to cell proliferation and tumor growth through generation of increased polyamines including not only putrescine but also the higher-order polyamines spermidine and spermine. The *ODC1* gene is a direct target of MYC and related MYCN and a *bona fide* oncogene that can transform cells[11–13]. Therefore, the inhibition of the polyamine pathway is considered an effective route for the treatment or prevention of cancers as confirmed by numerous studies examining the regulation of polyamine biosynthesis on cell proliferation and its inhibition by DFMO[9,14–21]. Previous work has confirmed the expression of ODC in the endometrium[22]. Furthermore, DFMO was found to inhibit growth of HEC-50 endometrial cancer cells *in vitro* a process that could be reversed following addition of putrescine[22]. A single study determined that ODC was present in increasing active amounts in normal, premalignant and malignant endometrium[23], however, insufficient numbers of cases in this study prevented any clinical associations[23]. These same authors noted decreased serum polyamine levels in some patients receiving conservative progestin treatment for their cancer[24]. Studies in the pig identified that cyclic progesterone regulated levels of IGF1 and Spermidine/spermine N^1^-acetyltransferase (SAT1) were critical in regulating the proliferative process of the endometrium[25]. Specifically, the normally growth suppressive activity of the catabolic enzyme SAT1, a key negative regulator of polyamine biosynthesis, was enhanced during the secretory phase (progesterone influence) while ODC levels were constant throughout the cycle.

Despite these early studies which pointed to a critical role for ODC and polyamine biosynthesis in endometrial proliferation and cancer there have not been concerted follow-up studies to rigorously examine the efficacy of DFMO in either the treatment or chemoprevention of primary or recurrent metastatic endometrial cancer. Here we describe the expression characteristics of *ODC1* in endometrial cancers and address the preclinical effects of DFMO on endometrial cancer *in vitro* and *in vivo*. Recent successes in repurposing this successful FDA-approved, orally available drug in the chemoprevention of colorectal cancer and pediatric neuroblastoma suggest its rapids translation and extended use in endometrial cancer patients[26,27].

## Materials and methods

### Cell culture

Cells were obtained from the following sources. EM-TERT cells were kind gift from Dr. Kyo the originator and described[28]. ACI-45, ACI-52, ACI-61, ACI-70, ACI-80, ACI-98, ACI-126, ACI-181 were established in the Risinger lab and previously described[29]. MSU-12 and MSU-15 were recently established (Risinger) and described here for the first time. MSU-12 cells were from a 66-year-old Caucasian patient’s drug treatment naive FIGO IIIC2 grade 3 uterine serous carcinoma. MSU-15 cells were established from a 58-year-old Caucasians drug treatment naive FIGO IIIC2 grade 3 mixed endometrioid and clear cell carcinoma. MSU-15 cells form predominantly clear cell tumors in immune deficient mice and the patient’s nodal metastasis were pure clear cell in histology. Long established endometrial cell line models HEC-1-A, KLE, and ECC-1 (variant of Ishikawa) were obtained from the American Type Culture Collection (Rockville MD). All cells were maintained in DMEM/F-12 media supplemented with 10% fetal bovine serum, penicillin, streptomycin and cultured in humidified 5% CO_2_ condition. D,L-alpha-difluoromethylornithine (DFMO) was dissolved in water and added at concentrations described for cell culture and animal studies and are consistent within the range of dosage levels delivered to animals and patients[27,30,31]. DFMO was provided by Dr. Patrick Woster (Medical University of South Carolina, Charleston, SC).

### Quantitative real-time PCR

Flash frozen samples were subject to total RNA isolation with TRIzol Reagent (Ambion, Thermo Fisher Scientific, Waltham, MA) according to manufacturer’s recommendation. Two μg of RNA were first treated with OPTIZYME DNase I (Thermo Fisher Scientific, Waltham, MA) for the preparation of DNA-free RNA prior to the transcription into complementary DNA (cDNA) with qScript cDNA Synthesis Kit (Quanta Biosciences, Beverly, MA). These cDNA were used as the template for the quantitative real-time PCR using primers with PerfeCTa SYBR Green FastMix reagent Quanta Biosciences (Beverly, MA) and the primers with the following sequences for *ODC1* forward 5’-CAGCTTTCACGCTTGCAGTT-3’ and reverse 5’-ATCTTCGTCATCAGAGCCCG-3’, *SAT1* forward 5’-GGCATAGGATCAGAAATTCTGAAGA-3’ and reverse 5’-CTGCTACCAAGAAGTGCATGCTG-3’, *SRM* forward 5’-CCCTCCGTGGAGTCCGTGGTC-3’ and reverse 5’-CTGGCAGGAACTTCTTGGAGACTTG-3’, *SMS* forward 5’-TGGAAATATTCTCATCCTTAGTGGG-3’ and reverse 5’-CGGGTATATGCCAAATCACTCTCT-3’, *PAOX* forward 5’-GGCGCACCATGTCATCGTCAC-3’ and reverse 5’-GGGGAGGGTCAAAGAAGGTGTCC AA-3’, *SMOX* forward 5’-CAATGCTGAAAGTCAAAATAGCGTG-3’ and reverse 5’-CTCTGGGTCGTCAGGGTC ATTCC-3’. All qPCR were done on Stratagene MX3000P and the mRNA quantities were normalized using Applied Biosystems human PPIA (cyclophilin A) endogenous control (Thermo Fisher Scientific, Waltham, MA).

### Cell viability assay

Cells were plated in triplicate at a density of 3,000 cells/well in a 96-well plate and treated with different concentrations of DFMO varying from 0.25 mM to 5 mM final concentration and different time points. A novel tetrazolium compound (MTS) and an electron coupling reagent (phenazine ethosulfate; PES) from The CellTiter 96^®^ AQueous One Solution Reagent (Promega, Madison, WI) was added to each well, then incubated for an hour and the absorbance read at 490 nm.

### Colony formation assay

Cells were plated at 3,000 cells/well in 6-well plate and allowed to grow over a two-week period with or without DFMO (1 mM). Resulting plates were analyzed with the crystal violet staining method. Briefly, cells were washed two times with ice-cold PBS then fixed with ice-cold methanol for 10 minutes. Enough 0.5% crystal violet solution was applied to each well to ensure full coverage of the area for a 10 minute time period at room temperature. Wells were then rinsed out with distilled water until all excess stain was removed. Plates were air dried overnight at room temperature. Stained colonies were counted the detected using imageJ software. All reagents were purchased from Thermo Fisher Scientific (Waltham, MA).

### Polyamine pool analysis

Polyamines (Put, Spd, Spm) from cell pellets, tumor tissues and mouse blood plasma were isolated, dansylated, and analyzed by HPLC as previously described[32]. Briefly, polyamines were extracted and protonated in perchloric acid/sodium chloride buffer. To 100 μl of sample, 4.5 nmol of 1,7 diaminoheptane internal standard and 200 μl of 1M sodium carbonate was added prior to dansylation with 400 μl of 5 mg/ml dansyl chloride (Sigma Aldrich, St. Louis, MO). Samples were analyzed using a Thermo Scientific/Dionex Ultimate 3000 HPLC equipped with a Syncronis C18 column (250 x 4.6 mm, 5 μM pore size). The dansylated polyamine derivatives were visualized by excitation at 340 nM and emission at 515 nM. Using the relative molar response derived from N-dansylated polyamine and 1,7 diaminoheptane standards, the amount of N-dansylated polyamine derivatives was calculated and normalized to total sample protein (cells, tumors) or starting volume (plasma).

### *In vivo* xenografts

Seven week old female athymic nude (Crl:NU(NCr)-*Foxn1*^nu^) mice were obtained from the Van Andel Research Institute (VARI) breeding colony. Tumor forming ability was assessed under an IACUC approved protocol XPA-16-07-005 “Treatment of endometrial cancer with DFMO” as part of VARI’s tumor biology core (Eagleson, PI). Mice were injected with buprenorphine (0.1 mg/kg subcutaneous) 30 minutes before initiation of the procedure. Mice were then flank-injected with 1 million ACI-98 cells suspended in a 1:1 mixture of PBS/Matrigel basement membrane C (Corning, Corning, NY). Time of injection was considered 0 days post injection (dpi). Mice were randomized to treatment groups (2% w/v DFMO or water alone, N = 10 animals per group) and daily monitored by the VARI preclinical therapeutics core staff that was blinded to treatment groups and anticipated outcomes. Volumes of drinking water were recorded for each group and no difference was found in consumption between the treatment group and controls. Mice did not develop any observable defect in overall appearance and activity level except the growing tumors which were measured by calipers starting at 10 dpi and recorded every 2 days thereafter. When the tumor size reached the maximum size threshold according to the IACUC protocol (2500 mm^3^), mice were euthanized with CO_2_ at 19 dpi, and blood collected, tumors removed and processed by the VARI mouse technical tumor staff. All mouse work complied with the requirements set forth in the Guide for the Care and Use of Laboratory Animals.

### Bioinformatic and biostatistical analyses

RSEM Normalized RNA-Seq (UNC Illumina HiSeq RNASeq V2, level 3) data was downloaded from the TCGA website. From these, 353 cases were considered for the analysis and their clinical parameters were taken from cBioportal for The Cancer Genome Atlas Uterine Corpus Carcinoma dataset[33–35]. Flash frozen prospective validation samples of endometrial cancer were obtained from Spectrum Hospital System recurrence and survival data and qRT-PCR results were analyzed from 60 samples. Laser captured endometrial cancers and glandular epithelium samples were also previously arrayed and published[36–38]. A subset (n = 132) of these 188 cancers had survival characteristics the clinical features for these samples are provided in S1 Table. Cox regression was performed considering proportional hazards model using survival package in R-environment for both overall survival (OS) and recurrence free survival (RFS). Affymetrix probesets used for analysis were; ODC1 (200790_at), SAT1 (203455_s_at), SRM (201516_at), SMS (202043_s_at), PAOX (221941_at) and SMOX (210357_s_at).

## Results

### *ODC1* is highly expressed in endometrial cancers

**Fig 1:**
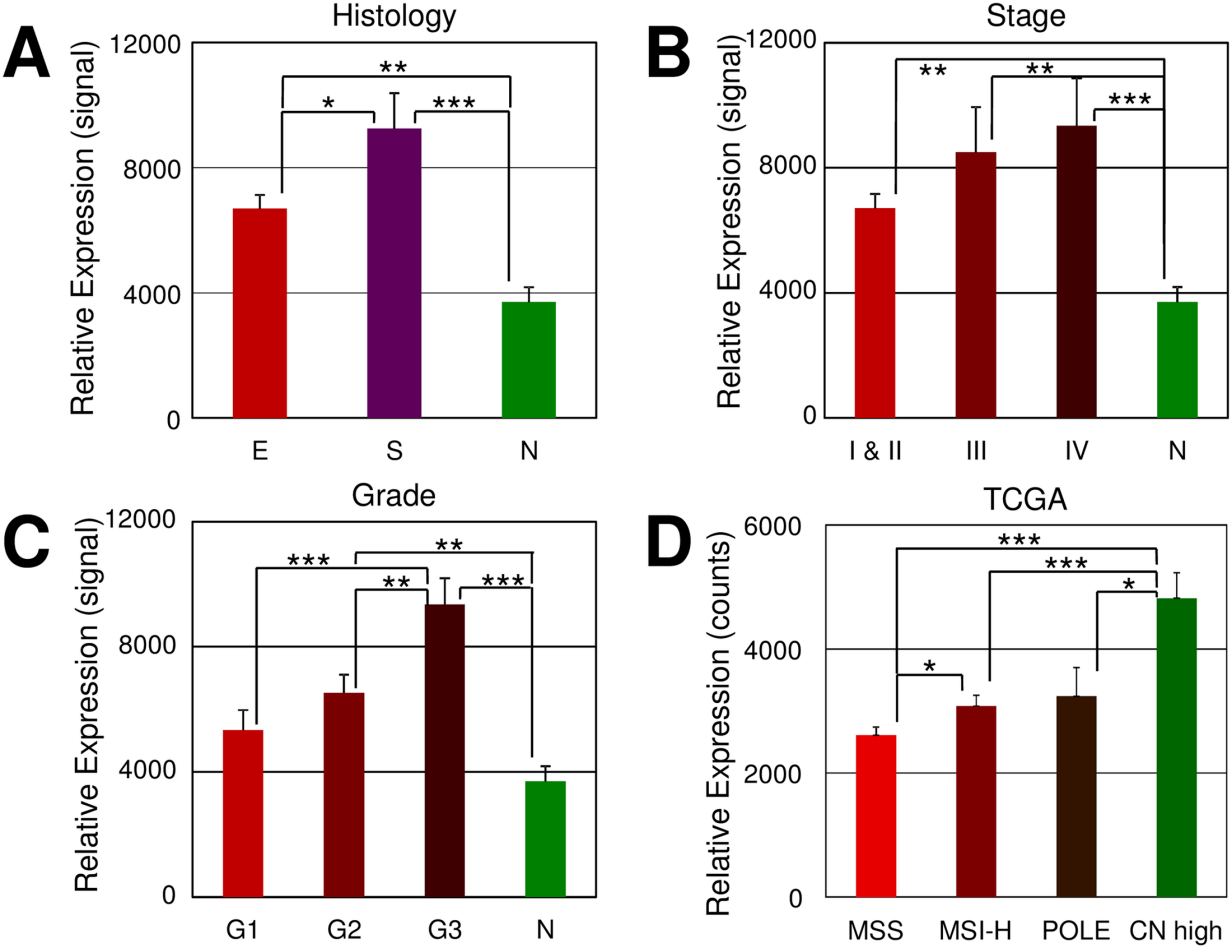
*ODC1* expression in endometrial cancers. A-C in LCM (laser capture micro-dissected) samples. **A**, Endometrioid (E, n = 139) and Serous (S, n = 37) types and normal epithelial tissues (N, n = 12); **B**, FIGO Stages I and II (n = 133), III (n = 24), and IV (n = 18); **C**, Grade 1 (G1, n = 42), Grade 2 (G2, n = 65), and Grade 3 (G3, n = 69). Signal: Affymetrix signal normalized to target value of 500. **D**, In TCGA samples of four molecular sub-types: Copy number low (MSS, n = 90), MSI high (n = 65), POLE ultra-mutant (n = 17) and Copy number high—serous like (n = 60). ***p < 0.001; **p < 0.01; *p< 0.05. Counts: Normalized RNA-seq counts.

While early studies suggested the importance of ODC and polyamines in regulating the growth of both normal and malignant endometrial cells there has been little follow-up and no comprehensive evaluation with the now known molecular underpinnings of this disease taken into account[22–25,39]. We have previously examined the transcriptome of laser captured endometrial cancers using Affymetrix gene chip technology[36,37]. We re-examined these expression data for probes specific for the principal genes in the polyamine biosynthesis pathway in data obtained from meticulously prepared laser captured samples with minimal stroma and immune infiltrates present. In our initial analysis we noted increased expression of *ODC1*, spermidine synthase (*SRM*) spermine synthase (*SMS*) and decreased expression of *SAT1* in cancers (n = 176) as compared to normal endometrial samples (n = 12) (S1 Fig). No significant changes were noted in polyamine oxidase (*PAOX*) and spermine oxidase (*SMOX*) expression between normal and cancer (S1 Fig). These data taken together suggest that the polyamine pathway is significantly activated in endometrial cancers in general. Because we found high *ODC1* expression we chose to focus on this important polyamine synthesis gene. We further explored the expression of *ODC1* in endometrial cancers noting significantly elevated expression in both endometrioid and serous cases the two main histotypes of endometrial cancer (Fig 1A and S1 Fig). We further noted the increased expression of *ODC1* in late stage (Fig 1B) and in higher grade cancers (Fig 1C) in these laser captured Affymetrix samples. Recently, molecular sub-types of endometrial cancer have been proposed to more accurately categorize this disease[33]. *ODC1* was expressed in all subtypes but we noted the significantly increased expression in the copy number high cancers (Fig 1D). This near ubiquitous expression confirms that ODC is a broadly applicable therapeutic target for this cancer type. Increased levels of *ODC1* were noted in high grade, late stage, serous histology or copy number high cancers which are all features associated with increased rates of recurrence and death in endometrial cancer[5,33].

### *ODC1* expression is associated with shorter time to recurrence and decreased survival

**Fig 2:**
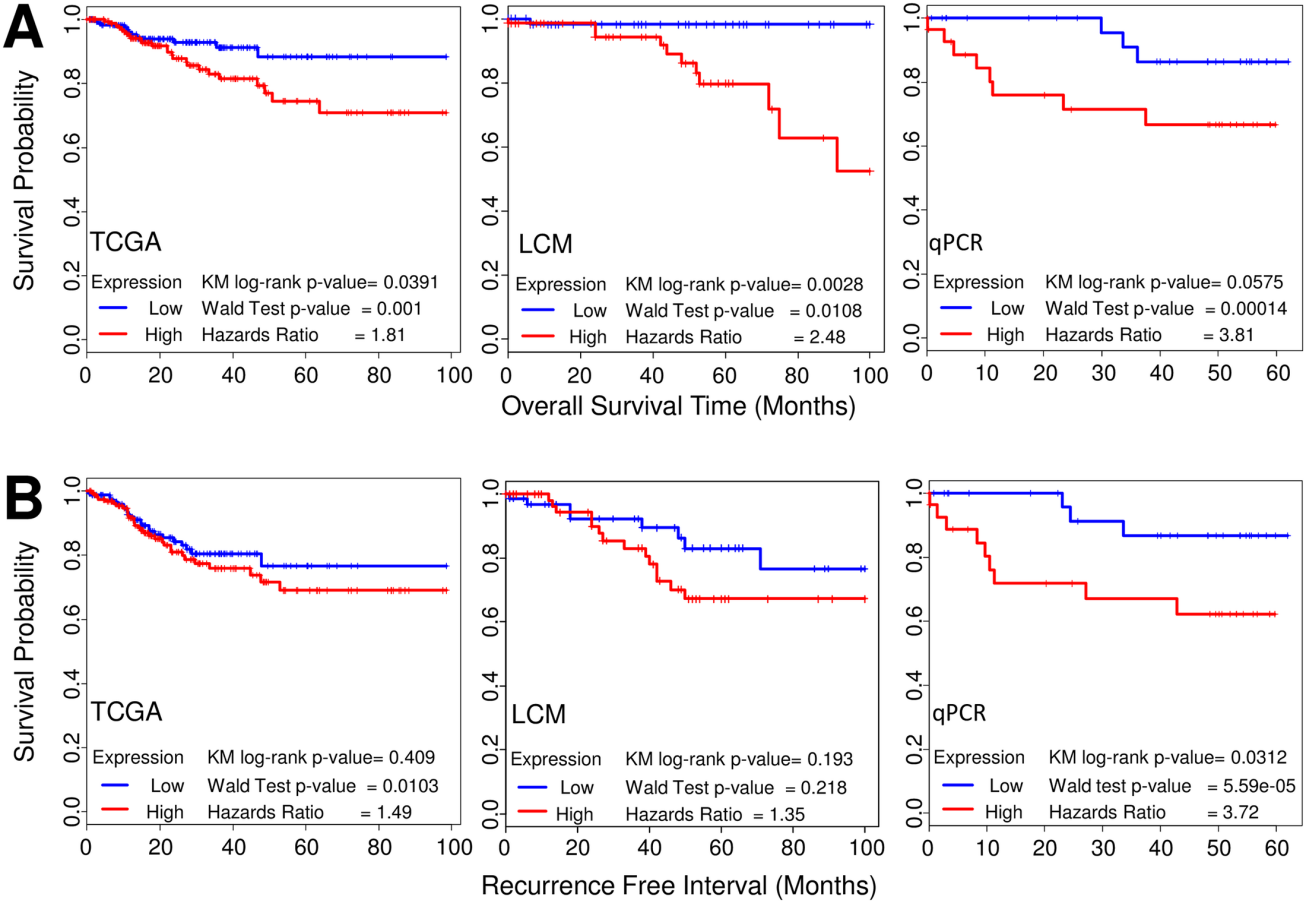
*ODC1* mRNA expression overall survival (OS) and recurrence free interval (RFI). Kaplan-Meier diagrams and Wald statistics showing increased *ODC1* is significantly associated with shorter OS (**A**) in TCGA (RNA-seq) (n = 232), LCM cases (Affymetrix) (n = 188) and in Spectrum (qRT-PCR) (n = 60) samples (**B**) *ODC1* mRNA is significantly associated with recurrence for TCGA and qRT-PCR cohorts but is not significant in the Affymetrix LCM dataset. “High” and “Low” indicate above and below the median expression level.

**Fig 3:**
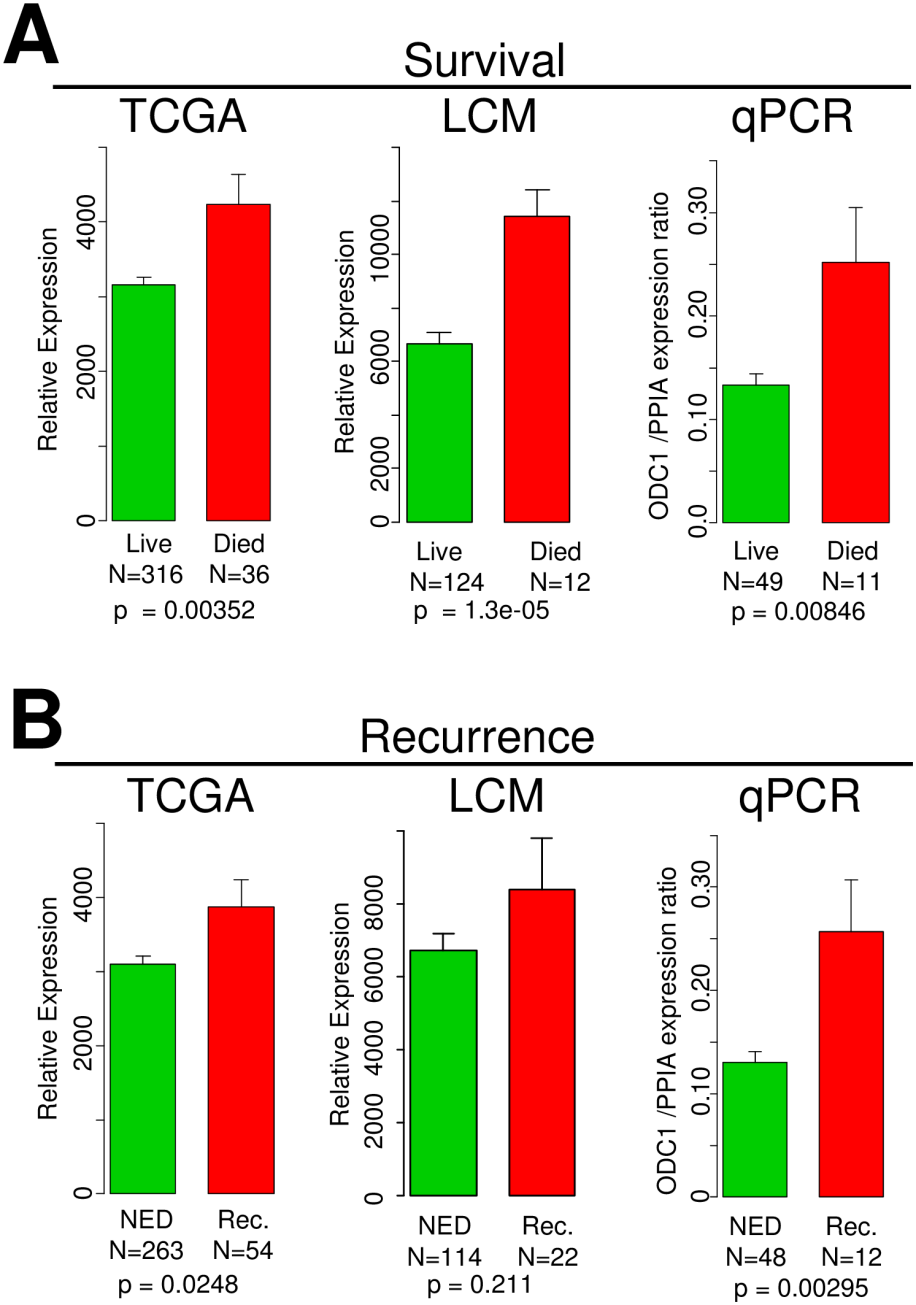
Bar graph of *ODC1* mRNA expression overall survival (OS) and recurrence free interval (RFI). **A**, Bar graph indicates relative expressions of OS in TCGA (RNA-seq) (p = 0.00352), LCM cases (Affymetrix) (p = 1.3e-05) and in Spectrum (qRT-PCR) (p = 0.00846) samples. **B**, Relative expressions of RFI in TCGA (RNA-seq) (p = 0.0248), LCM cases (Affymetrix) (p = 0.211) and in Spectrum (qRT-PCR) (p = 0.00295) samples. Rec.: Recurrence; NED: No Evidence of Disease.

The elevated levels of *ODC1* mRNA noted in those cancers with poor prognostic factors prompted us to directly examine whether *ODC1* mRNA levels themselves were prognostic for recurrence or survival. Therefore, we explored the association of *ODC1* expression with clinical outcomes of overall survival (OS) and recurrence in the published TCGA cohort and noted that elevated *ODC1* was significantly related to OS (KM log-rank p = 0.0391, Wald test p = 0.001) and recurrence (Wald test p = 0.0103) (Figs 2 and 3). Levels of *ODC1* were also associated with worse OS in our LCM Affymetrix dataset (Fig 2A). Importantly, we confirmed these observations using a validation cohort of 60 endometrial cancers with well annotated clinical follow-up using quantitative PCR assays for *ODC1*. These data also indicated that endometrial cancers with elevated *ODC1* had significantly shorter recurrence-free intervals (KM log-rank p = 0.0312, Wald test p = 5.59e-05) and an elevated hazard ratio 3.72 (Fig 2B). Similar to the TCGA and Affymetrix cohorts we also noted an association worse OS (KM log-rank p = 0.0575, Wald test p = 0.00014) with elevated hazard ratio 3.81 (Fig 2A). Taken together these three datasets independently indicate that elevated *ODC1* is associated with shorter time to recurrence and decreased survival times. In all datasets expression of *ODC1* was higher in those cases with reduced survival and shorter time to recurrence (Fig 3).

### DFMO inhibits growth of endometrial cancer cells *in vitro*

**Fig 4:**
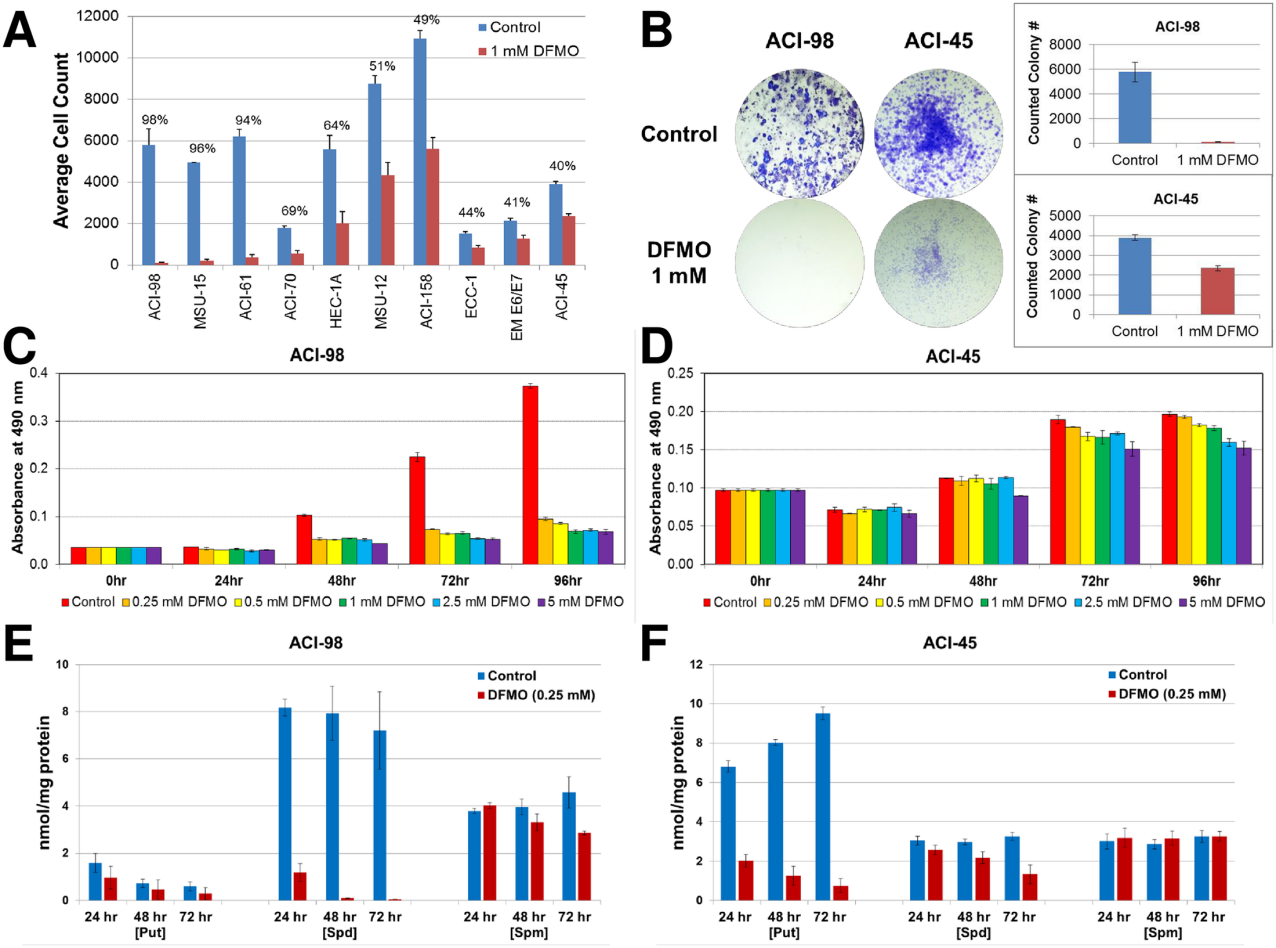
Colony formation, cell viability and polyamine levels in DFMO-treated endometrial cancer cells. **A**, Colony counts in a panel of endometrial cancer and immortal normal endometrial epithelial cells including ACI-98 (undifferentiated), MSU-15 (clear cell), ACI-61 (endometrioid) ACI-70 (MMT), HEC-1-A (endometrioid), EM E6/E7 TERT1 (normal immortalized endometrial epithelial), ECC-1 (endometrioid) and ACI-45 (carcinosarcoma, MMT). Cells are ordered most highly sensitive on left to least sensitive on the right. **B**, Representative colony plates for most (ACI-98) and least (ACI-45) sensitive cells. **C** and **D**, ACI-98 cells highly sensitive to wide range of doses of DFMO while ACI-45 cells are non-sensitive even at very high doses (5 mM) as measured by MTS assay. The percent values (%) shown are DFMO to Control. Intracellular polyamine levels (putrescine, spermidine, spermine) were analyzed in ACI-98 (**E**) and ACI-45 (**F**) cells either untreated (control) or DFMO-treated (0.25 mM) using RP-HPLC. Intracellular polyamines were quantified and expressed as nmol/mg protein. Standard errors are indicated (+/- S.E.). Put, putrescine; Spd, spermidine; Spm, spermine.

We rigorously tested the ability of DFMO to inhibit growth in a panel of uterine cancer cell lines which cover most important histotypes and feature different underlying driver mutation profiles. Cell colony viability studies indicated that endometrial cancer cells ACI-98 (undifferentiated), MSU-15 (clear cell), ACI-61 (endometrioid) ACI-70 (carcinosarcoma) and HEC-1-A (endometrioid) were highly sensitive to the anti-proliferative effects of DFMO at dose ranges utilized in human clinical studies. In contrast, EM E6/E7 TERT1 (normal immortalized endometrial epithelial cells), ECC-1 (endometrioid) and ACI-45 (carcinosarcoma) were less sensitive (Fig 4A). Although most of these cells including ACI-98 showed significant inhibition, some cell models, most notably the immortal normal endometrium and ACI-45, showed far less response to DFMO (Fig 4B). We further examined cell viability in ACI-98 and ACI-45 and found ACI-98 cells were highly sensitive to DFMO even at 0.25 mM doses whereas ACI-45 cells still retained greater than 75% viability even at 5 mM DFMO (Fig 4C and 4D). We examined mRNA levels of *ODC1*, *SAT1*, *SRM*, *SMS*, *PAOX* and *SMOX* and noted expression levels of these key polyamine pathway genes in all cell lines (S2 Fig). We examined cellular polyamine levels in untreated and 0.25 mM treated ACI-98 and ACI-45 cells using reverse phase high performance liquid chromatography (RP-HPLC). We were able to document significant levels of putrescine, spermidine, and spermine in both cell models with ACI-98 expressing 3-fold higher levels of spermidine than ACI-45, whereas ACI-45 expressed higher levels of putrescine (Fig 4E and 4F). Low dose (0.25 mM) DFMO significantly reduced spermidine levels in ACI-98 cells approximately 5-fold by 24 hours and essentially extinguished measurable spermidine at 48 and 72 hours (Fig 4E and 4F). DFMO treatment significantly reduced putrescine levels in ACI-45 cells but levels of spermidine remained refractory to DFMO treatment at 24 hours and only slightly reduced at later time points. Spermine levels were not affected in ACI-45 cells. Similarly spermine levels were unchanged in early 24 hour time points and only slightly reduced in ACI-98 cells at later time points (Fig 4E and 4F). These *in vitro* data strongly suggest that a subset of endometrial cancers are addicted to polyamines and exhibit a high degree of sensitivity to polyamine pathway inhibition.

### DFMO treatment significantly reduced endometrial tumor growth *in vivo*

**Fig 5:**
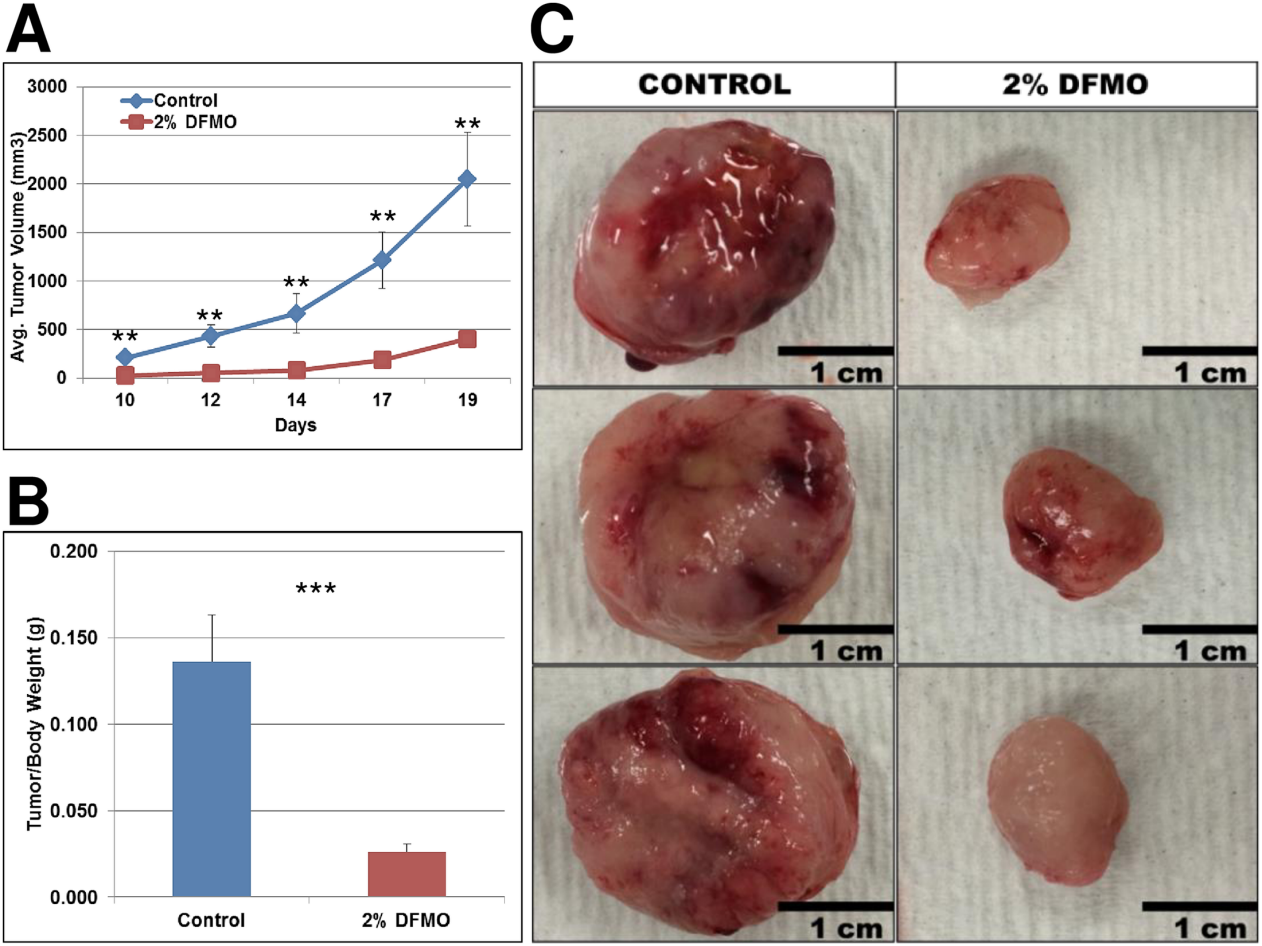
Oral DFMO significantly reduces tumor burden in tumor-bearing mice. Female athymic nude mice bearing xenografted endometrial tumors (ACI-98) received 2% (w/v) DFMO in drinking water or control (water only). Both control and DFMO-treated mice consumed similar quantities of water per day. Quantification of tumor volume (**A**) and tumor weight (**B**) of DFMO-treated and untreated tumors. Ten mice per group (n = 10). **C**, Representative images of DFMO-treated and untreated tumors.

**Fig 6:**
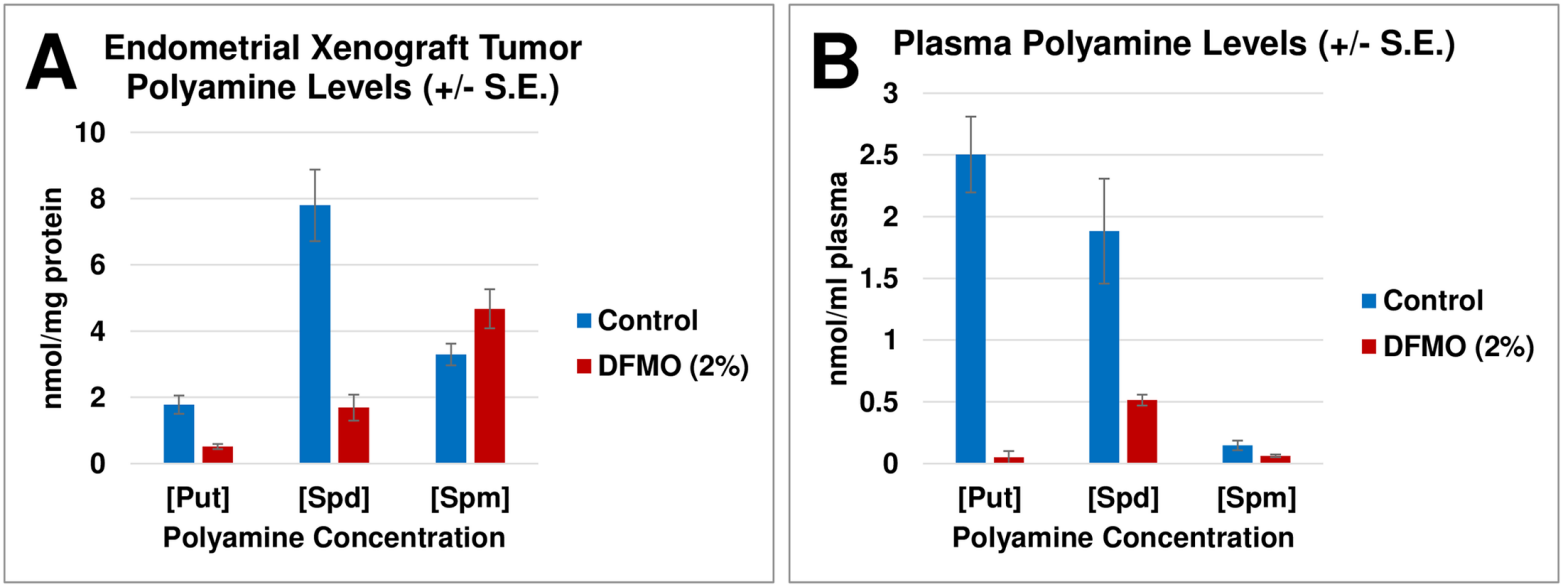
Effect of oral DFMO on polyamine levels in tumor-bearing mice. Intracellular polyamine levels (putrescine, spermidine, spermine) were analyzed in tumor tissue (**A**) and blood plasma (**B**) of DFMO-treated and untreated mice using reverse-phase HPLC. Intracellular polyamines were quantified and expressed as nmol/mg protein (**A**) or nmol/ml plasma (**B**). Standard errors are indicated (+/- S.E.). Put, putrescine; Spd, spermidine; Spm, spermine.

To confirm the *in vitro* data, we performed an *in vivo* study with human endometrial tumor-bearing mice to verify whether significant effects of DFMO seen *in vitro* using a panel of endometrial cancer cell lines would also be seen *in vivo*. We chose to examine the effects of DFMO on the growth of ACI-98 cell *in vivo*. ACI-98 cells are highly tumorigenic in nude mice. Nude mice were inoculated with 1 million ACI-98 cells contained in a bolus of matrigel and mice were either treated with 2% (w/v) DFMO supplied in drinking water or water only (n = 10/group) for 19 days. Tumor sizes were measured every 2 days starting at day 10 and large tumor sizes in untreated mice required sacrifice for untreated cohort at 19 days. DFMO treatments were found to significantly reduce the tumor burden in mice compared to controls with tumor volumes and weights both significantly decreased in DFMO treated animals (Fig 5). We harvested tumor tissues and plasma at the end of the study and examined tumor and plasma levels of polyamines using reverse phase high performance liquid chromatography (RP-HPLC). Importantly, we noted that the ODC-regulated polyamines (putrescine, spermidine) known activators of cell proliferation were strongly decreased in mice treated with DFMO, in both tumor tissue (Fig 6A) and blood plasma (Fig 6B). The reduction of these surrogate markers confirms that the effect of DFMO is specific and targets ODC by blocking the polyamine biosynthetic pathway. No significant changes were observed in the tissue or plasma spermine levels. The effects of DFMO on putrescine (a diamine) and spermidine (a triamine) with varying/minimal effects on the higher-order polyamine spermine (a tetramine) have previously been reported and can be explained by the fact that frequently, cell proliferation is halted before significant depletion of the polyamine spermine can occur[40,41].

### Polyamine metabolism in uterine cancers

The complexity of polyamines metabolism and tightly regulated homeostasis support its importance for cell maintenance and function. Thus any abnormalities in the polyamines metabolism might implicate several pathological processes such as cancers. Since we here investigate *ODC1*, a key polyamine regulator in endometrial cancers, we decided to also examine other essential enzymes involved in polyamine metabolism in order to address their significance in the disease. Affymetrix LCM dataset analysis on uterine endometrial cancers and uterine serous cancers showed an elevated cancer/normal expression ratio with biosynthetic enzymes such ODC1, spermidine synthase (SRM) and spermine synthase (SMS), while the ratio of catabolic enzymes like SAT1, polyamine oxidase (PAOX) and spermine oxidase (SMOX) showed no difference in expression (S2 Fig). These data are in support of our *in vitro* and *in vivo* analyses that suggest endometrial cancers with elevated polyamines might respond sensitively to polyamine pathway inhibition.

## Discussion

In this study we examined in depth the expression characteristics of ODC in endometrial cancer. We found *ODC1* is widely expressed across different histologic types of endometrial cancer as compared to normal endometrial epithelium. Importantly, we noted elevated expression in those cancers with established features of poor prognosis for this cancer type. Specifically, highest expression of ODC was seen in late stage, grade 3, serous histotypes and in those copy number high molecular classified cancers. Elevated ODC expression is often linked with increased MYC activity in cancers[11,12,16,31]. *MYC* gene amplification and high MYC expression are common events in some high-grade endometrial cancers and this is linked to worse survival characteristics[7,42–45]. We used cBioPortal and queried endometrial cancers present in the published TCGA cohort and found that those with copy number amplification or gain and elevated expression of MYC also had elevated *ODC1* (p = 0.02) (data not shown). We also examined DNA gain or amplification across *MYC*, *MYCN* and *MYCL* in the TCGA endometrial cancer cohort[33–35]. These data showed that gain or amplification occurred in *MYC* in 28%, *MYCN* in 17% and *MYCL* in 7%. Cancers with gain or amplification in one *MYC* gene were also likely to have gain in more than one *MYC* gene (p<0.001) and more than 1/3 of cancers had increased DNA copy number change in at least one *MYC* gene. Amplification and overexpression of *MYCN* has not been described for human uterine cancer but was noted along with *ODC1* as being amplified in rat endometrial cancer[46]. Of note, *ODC1* which is located within 5 Mb of *MYCN* is co-amplified with *MYCN* in 15–20% of *MYCN*-amplified neuroblastomas and *ODC1* is significantly overexpressed in those tumors, thus presenting the first example of oncogene-target gene co-amplification[47]. *ODC1* itself was amplified in only 3% of cancers and had copy number gain in 13% often in the same cancers with *MYC* amplifications. Given the previously established role of MYC and *ODC1* promoter activity it is likely that these DNA copy number gains contribute to the increased ODC activity in these particular cancers especially high grade cancers where DNA copy number change is more frequently present. However, we noted high expression of *ODC1* in the absence of *MYC* amplifications suggesting other mechanisms may also be contributing to the observed increases in ODC. Recent work has shown a role for the progesterone receptor in down-regulating MYC in endometrial cancers[48]. Future studies should clarify the role of MYC and ODC in endometrial cancers.

In addition, to the detailed description of *ODC1* expression across endometrial cancer subtypes and normal endometrial cancers we also noted significant changes in the expression of other key regulators of polyamines in endometrial cancers as compared to normal. Specifically we noted down-regulation of *SAT1* expression across both endometrioid and serous histotypes. Some previous studies suggested that progestin regulated SAT1 modulated polyamine levels in the uterus. Reduced levels of SAT1 in endometrial cancers would suggest this may reflect a loss of ovarian steroid anti-proliferative controls which is the hallmark feature of endometrial cancer. We also noted significant upregulation of spermidine synthase (SRM) and spermine synthase (SMS) enzymes which further process polyamines into spermidine and spermine, respectively, while the polyamine oxidase (PAOX) and spermine oxidase (SMOX) enzymes showed no difference of expression from normal endometrium. Our *in vitro* studies suggested that endometrial cancers may not equally respond to the anti-proliferative effects of DFMO. Expression of polyamine pathway genes were not very informative in predicting which cells might be more or less sensitive *in vitro* based on expression of these key genes. For example, levels of *ODC1* were extremely high in ACI-61 one of the 2 most sensitive cell models but these ODC levels were confounded by equally high levels of SAT1. Therefore, it is unclear whether mRNA or protein levels of these genes/enzymes are all that relevant under the conditions of the cell culture where cell media provides copious exogenous polyamines. Future studies should explore the role of SAT1, SMS and SRM in the polyamine addiction of endometrial cancer, in particular the mechanisms that cause some of these to be more DFMO sensitive than others.

We chose to re-examine the role of the polyamine pathway in endometrial cancers for several reasons. Firstly, there was a paucity of work on this cancer type as compared to other types. While these early works were suggestive of a critical role in driving these cancers it was not fully explored. More importantly, however, is the increasing burden of endometrial cancer in developed countries. Unlike many other cancers which are decreasing in incidence or maintaining current levels in the U.S., the numbers of endometrial cancers is increasing as is the death rate[1,2].

Endometrial cancer is a heterogeneous disease. Advanced stage, high tumor grade, non-endometrioid histology and absence of estrogen and progesterone receptors are associated with recurrence and death[7,8]. Endometrial cancers are characterized by frequent mutations in oncogenes and tumor suppressors and tumors display different mutator phenotypes[7]. DNA mutation frequency and copy number changes have been proposed to divide endometrial cancers into 4 main molecular groups[33]. These groups are copy number low, typically low grade endometrioid histotypes that are microsatellite stable (MSS), with near diploid genomes and frequent *PTEN*, *KRAS*, *ARID1A*, *CTNNB1* and *AKT* pathway mutations, copy number high (CNH) high grade cancers with frequent genomic gain and loss typified by serous histology and *TP53* mutation, the hyper-mutated microsatellite instability-high (MSI-H) group of endometrioid type histology with a defect in DNA mismatch repair, and an ultra-mutated group characterized by high grade endometrioid cancers with defects in the polymerase epsilon (*POLE)* gene exonuclease domain (Ultra-mutant POLE). Both MSI and POLE mutant tumors harbor a similar mutation spectrum as other endometrioid cancers but with mutations at more elevated levels[7]. In terms of recurrence and survival, copy number high has the highest rates of recurrence and death whereas the POLE ultra-mutated group the most favorable survival[7,33]. We noted highest levels of *ODC1* expression in CNH tumors that have the worst prognosis.

The therapeutic effects of DFMO have not been extensively assessed in solid tumors. Levin *et al* have demonstrated an incremental survival advantage for analplastic glioma patients receiving DFMO with procarbazine/lomustine/vincristine (PCV) versus PCV alone giving median survival 6.3 years versus 5.1 years respectively[49]. Although DFMO has been shown to be well tolerated in Phase I trials involving prostate cancer patients, measurement of response has been limited given the small numbers of patients reported[50]. More recent investigations of DFMO as an intervention have focused on its utility as a chemopreventive agent[19,26,51–55]. Two prevention trials have demonstrated a reduction in polyps in populations of adenomato sis polyposis patients as well as patients with sporadic colonic polyps have demonstrated reduction in polyps in response to DFMO[26,56]. Even at very high doses DFMO has an exceptionally low propensity for side effects. Based on these findings, we could envision DFMO as an adjuvant therapy for women who have had surgical intervention for their endometrial cancer. Additionally, there is an unmet opportunity to prevent endometrial cancer metastasis or recurrence in those women who are apparently disease free following initial treatments yet may still suffer later recurrence and metastasis with adverse outcome.

The expression of *ODC1* across all molecular sub-types of endometrial cancer suggests targeting ODC would benefit all women requiring chemoprevention. Importantly, our finding that recurrent cancers have more *ODC1* suggests our DFMO-based chemopreventive approach would have particular merit for those cancers that ultimately cause death in patients with endometrial cancer. Additional chemopreventive opportunities in endometrial cancer exist for those Lynch syndrome family members who inherit a defective DNA mismatch repair gene and are at an extremely high risk for developing endometrial cancer in addition to colon cancers and other cancers in this syndrome. DFMO often in combination with NSAIDs has been successful in some trials at preventing polyps[26,54,55,57]. It is easy to envision prevention in this cohort of high risk women. However, recent findings that NSAID use may also increase endometrial cancer recurrence would suggest that inclusion of NSAIDs in such a regimen might be unwise[58].

## Conclusion

In summary our findings indicate that endometrial cancers are likely to be highly dependent on polyamines and susceptible to polyamine pathway inhibition. Cancers with highest levels of ODC are also those with the greatest need for new therapies as these are more likely to have tumor recurrence or patient death. Our preclinical work shows high sensitivity to DFMO both *in vitro* and *in vivo* suggesting that therapies specifically targeting ODC might have particular merit for some patients with high-risk endometrial cancers.

## Supporting information

S1 Fig Polyamine biosynthesis pathway gene expression in normal endometrial epithelium and cancer. LCM (laser capture micro-dissected) samples. **A**, Endometrioid (E, n = 139) and **B**, Serous (S, n = 37) types and normal epithelial tissues (N, n = 12); for key polyamine synthesis genes.
(PPTX)

S2 Fig Polyamine biosynthesis pathway gene expression in endometrial cell lines. Expression of key polyamine synthesis genes in endometrial cancer and immortal normal endometrial epithelial cells as measured by quantitative PCR. Endometrial cells including ACI-98 (undifferentiated), MSU-15 (clear cell), ACI-61 (endometrioid) ACI-70 (MMT), HEC-1-A (endometrioid), EM E6/E7 TERT1 (normal immortalized endometrial epithelial), ECC-1 (endometrioid) and ACI-45 (carcinosarcoma, MMT). Expression is relative to cyclophillin A (PPIA).
(PPTX)

S3 Fig Flow chart of animal study design. (PPTX)

S1 Table Dataset for LCM, TCGA and Spectrum samples. (XLSX)

S1 Checklist Completed ‘‘The ARRIVE Guidelines Checklist” for reporting animal study of the manuscript. (DOCX)
