# Supplementary figures and images for: Ornithine decarboxylase as a therapeutic target for endometrial cancer
Source: PLoS One. 2017 Dec 14;12(12):e0189044. doi: 10.1371/journal.pone.0189044 (PMC5730160; doi:10.1371/journal.pone.0189044)

## Slide 1
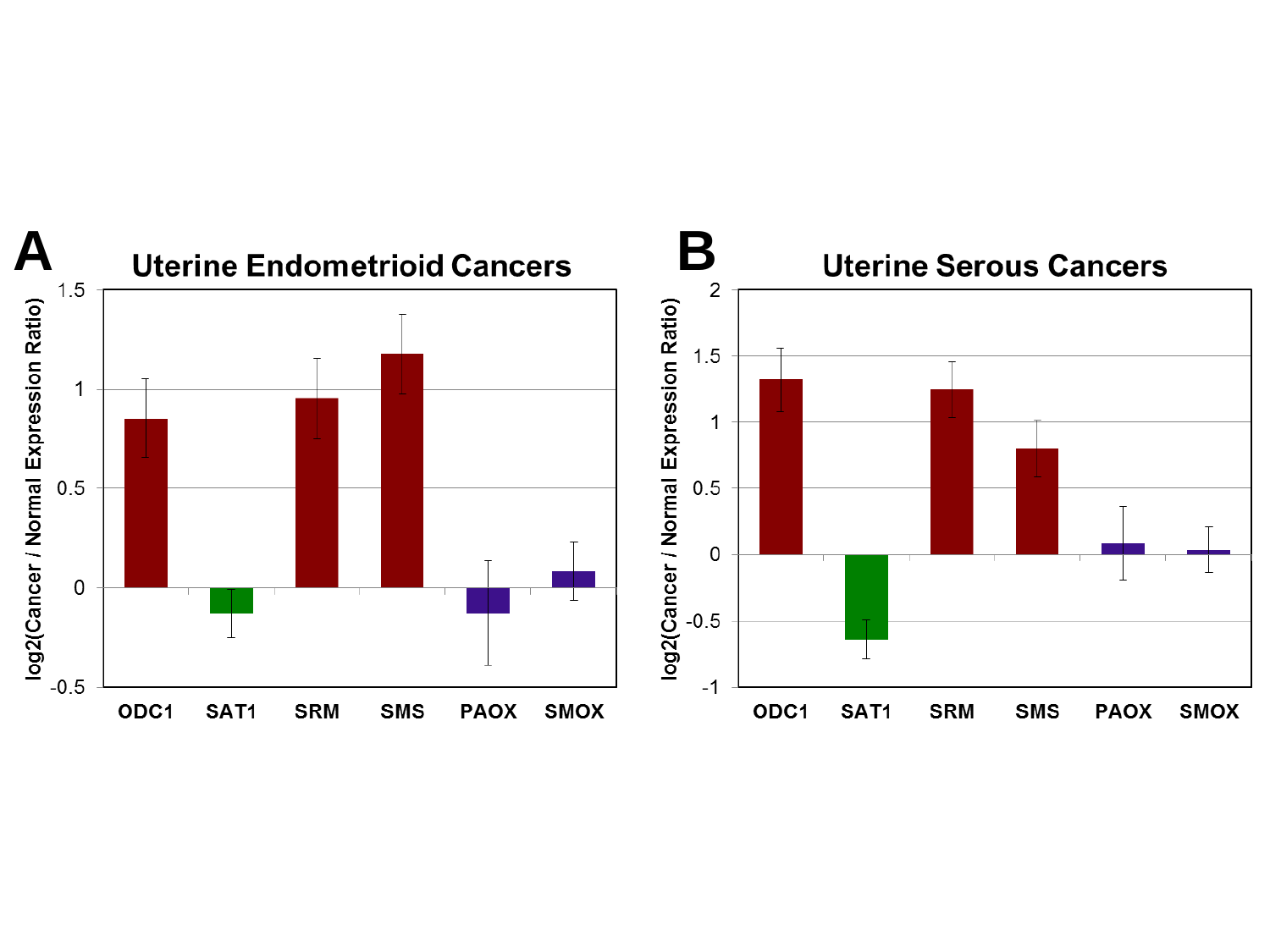

A
B

Supplement: S1 Fig — LCM (laser capture micro-dissected) samples. A, Endometrioid (E, n = 139) and B, Serous (S, n = 37) types and normal epithelial tissues (N, n = 12); for key polyamine synthesis genes. (PPTX) [file pone.0189044.s001.pptx]

## Slide 1
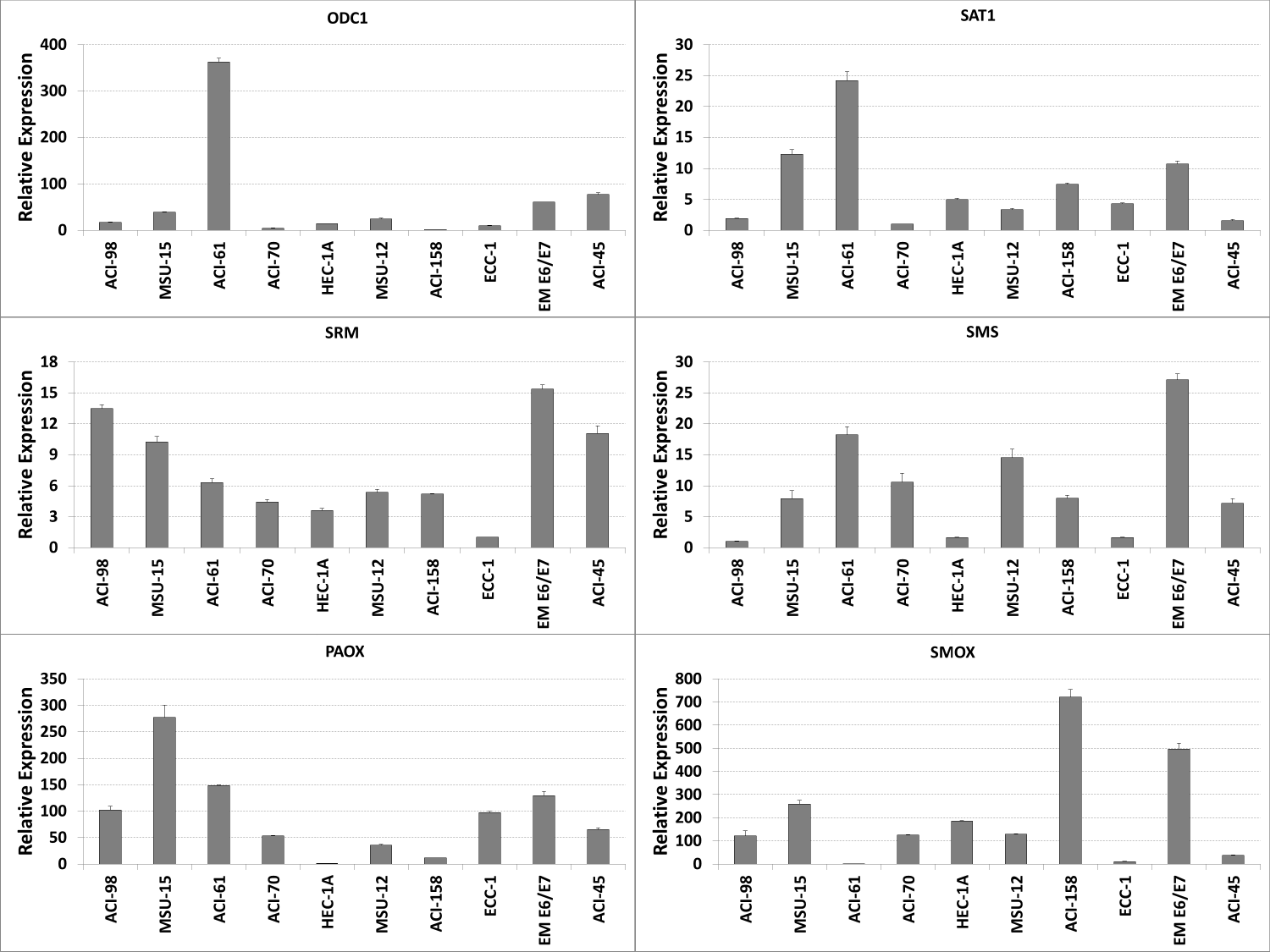

Supplement: S2 Fig — Expression of key polyamine synthesis genes in endometrial cancer and immortal normal endometrial epithelial cells as measured by quantitative PCR. Endometrial cells including ACI-98 (undifferentiated), MSU-15 (clear cell), ACI-61 (endometrioid) ACI-70 (MMT), HEC-1-A (endometrioid), EM E6/E7 TERT1 (normal immortalized endometrial epithelial), ECC-1 (endometrioid) and ACI-45 (carcinosarcoma, MMT). Expression is relative to cyclophillin A (PPIA). (PPTX) [file pone.0189044.s002.pptx]
