# Supplementary material for: Ornithine decarboxylase as a therapeutic target for endometrial cancer
Source: PLoS One. 2017 Dec 14;12(12):e0189044. doi: 10.1371/journal.pone.0189044 (PMC5730160; doi:10.1371/journal.pone.0189044)
Supplement: S3 Fig — (PPTX) [file pone.0189044.s003.pptx]

## Slide 1
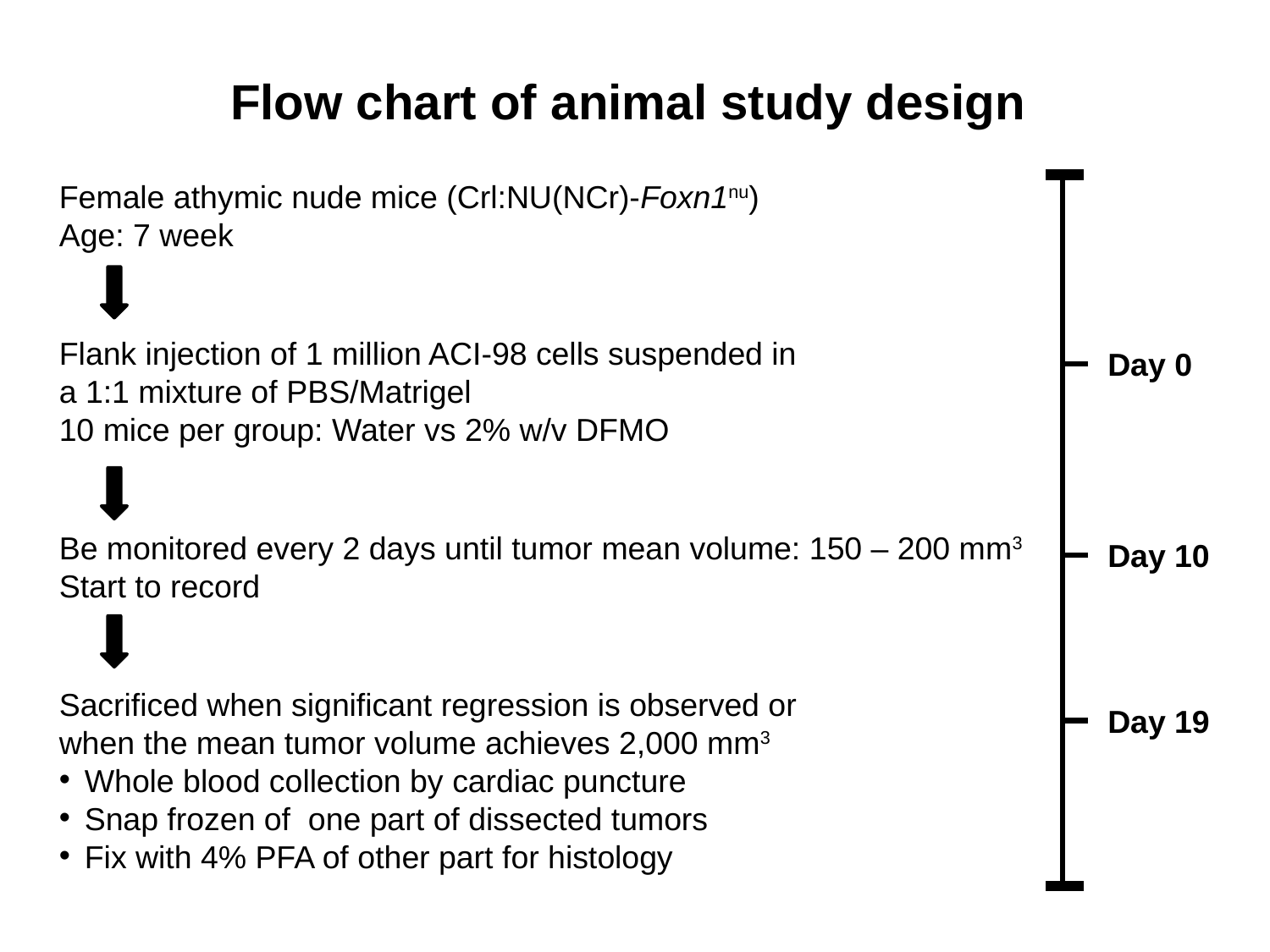

Flow chart of animal study design
Female athymic nude mice (Crl:NU(NCr)-Foxn1nu)
Age: 7 week
Flank injection of 1 million ACI-98 cells suspended in
a 1:1 mixture of PBS/Matrigel
10 mice per group: Water vs 2% w/v DFMO
Be monitored every 2 days until tumor mean volume: 150 – 200 mm3
Start to record
Sacrificed when significant regression is observed or
when the mean tumor volume achieves 2,000 mm3
Whole blood collection by cardiac puncture
Snap frozen of one part of dissected tumors
Fix with 4% PFA of other part for histology
Day 0
Day 10
Day 19
