# Supplementary material for: Ornithine decarboxylase as a therapeutic target for endometrial cancer
Source: PLoS One. 2017 Dec 14;12(12):e0189044. doi: 10.1371/journal.pone.0189044 (PMC5730160; doi:10.1371/journal.pone.0189044)

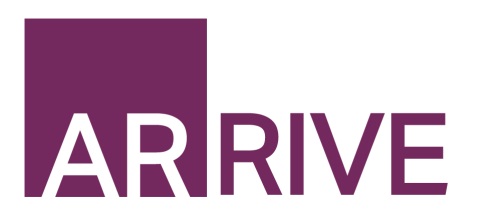


The ARRIVE Guidelines Checklist

Animal Research: Reporting In Vivo Experiments

Carol Kilkenny^1^, William J Browne^2^, Innes C Cuthill^3^, Michael Emerson^4^ and Douglas G Altman^5^

*^1^The National Centre for the Replacement, Refinement and Reduction of Animals in Research, London, UK, ^2^School of Veterinary Science, University of Bristol, Bristol, UK, ^3^School of Biological Sciences, University of Bristol, Bristol, UK, ^4^National Heart and Lung Institute, Imperial College London, UK, ^5^Centre for Statistics in Medicine, University of Oxford, Oxford, UK.*

|  | | ITEM | RECOMMENDATION | Section/ Paragraph |
| --- | --- | --- | --- | --- |
| 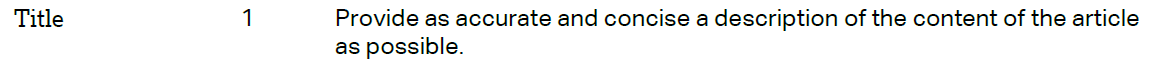 | | | Title |  |
| 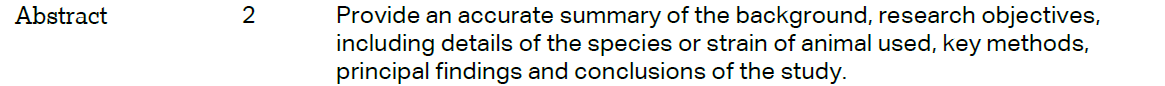 | | | Abstract |  |
| INTRODUCTION | | |  |  |
| 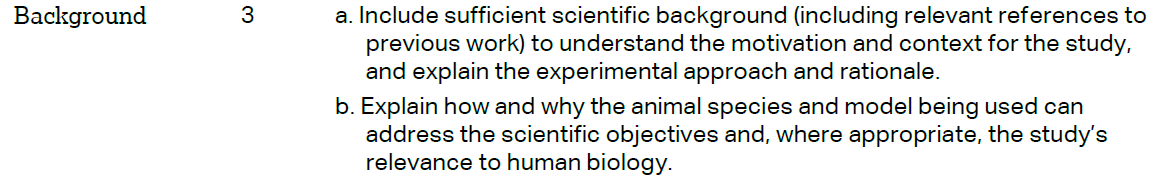 | | | Introduction: paragraph 1-4 |  |
| 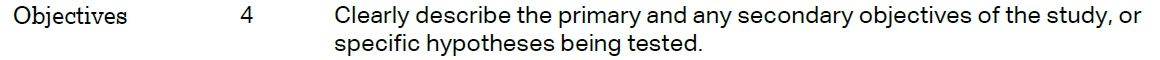 | | | Introduction: paragraph 4 |  |
| METHODS | | |  |  |
| 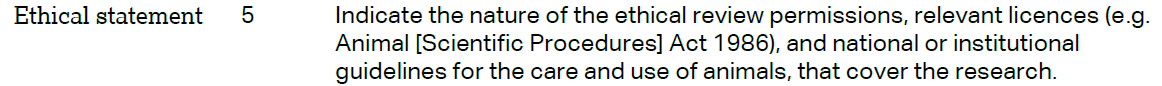 | | | Material and Methods: *In vivo* xenografts |  |
| 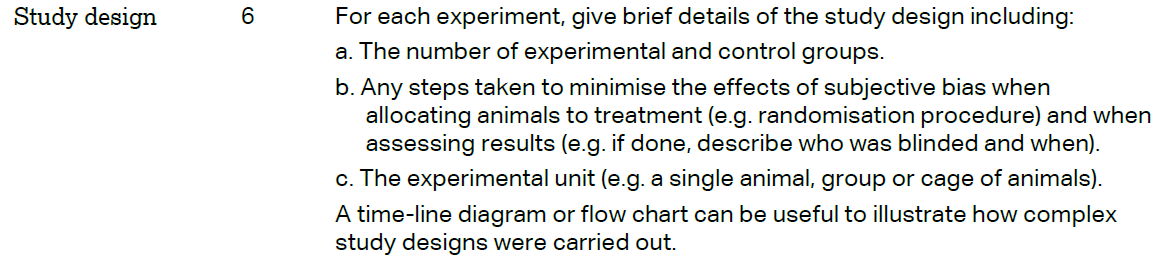 | | | *In vivo* Xenografts  S3 Fig |  |
| 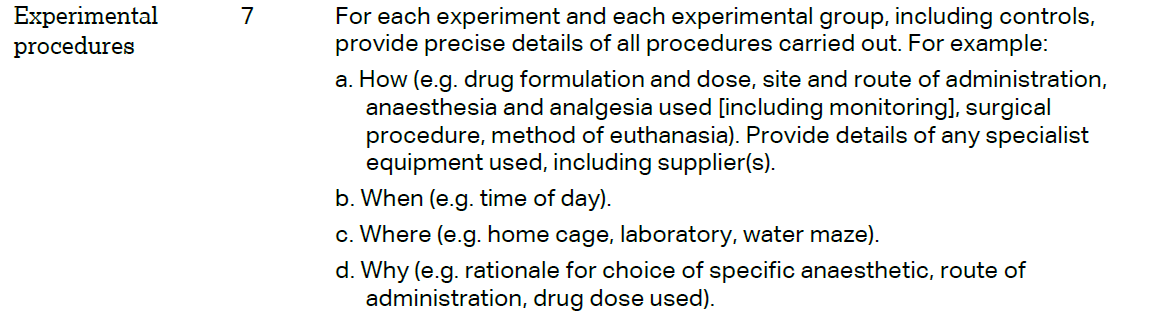 | | | *In vivo* Xenografts |  |
| 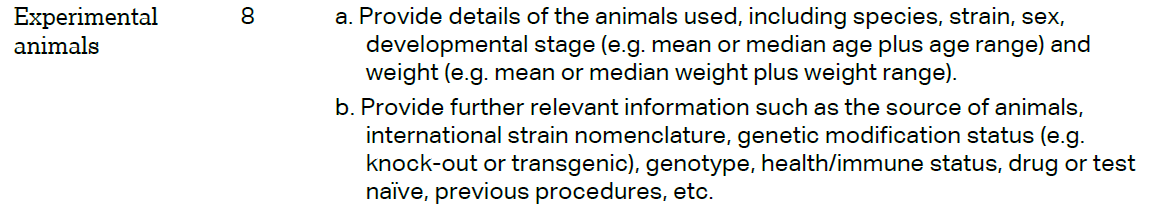 | | | *In vivo* Xenografts |  |

The ARRIVE guidelines. Originally published in *PLoS Biology*, June 2010^1^

| 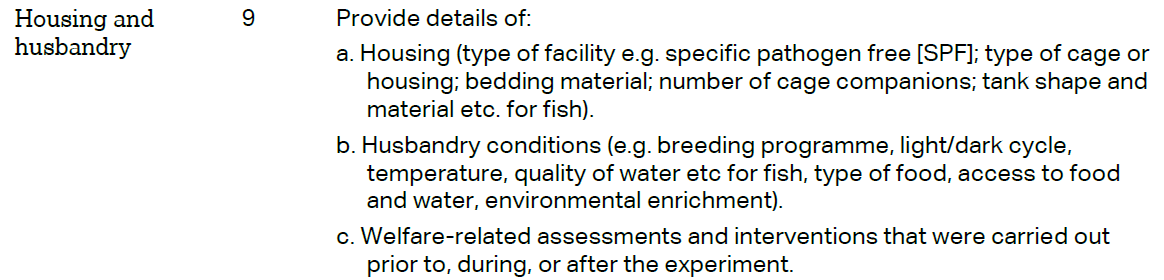 | *In vivo* Xenografts | |
| --- | --- | --- |
| 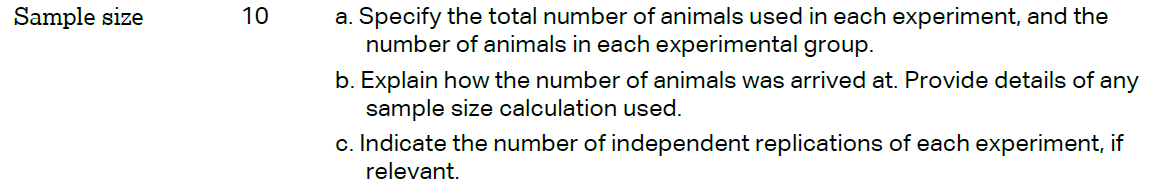 | *In vivo* Xenografts | |
| 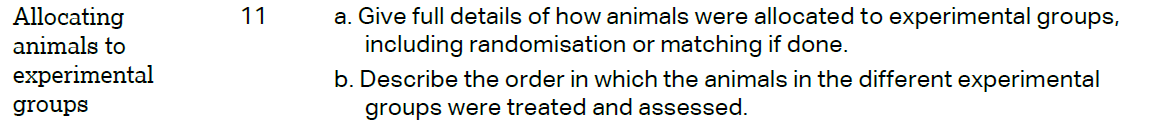 | *In vivo* Xenografts | |
| 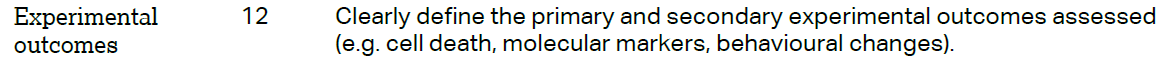 | *In vivo* Xenografts | |
| 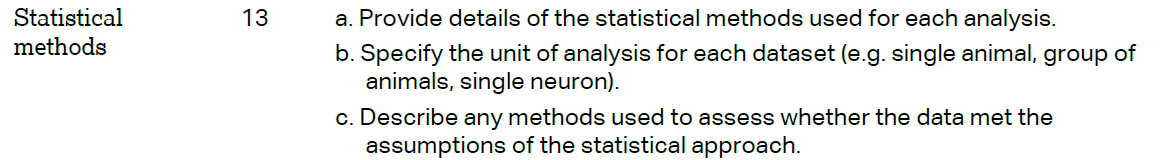 | *In vivo* Xenografts | |
| RESULTS |  | |
| 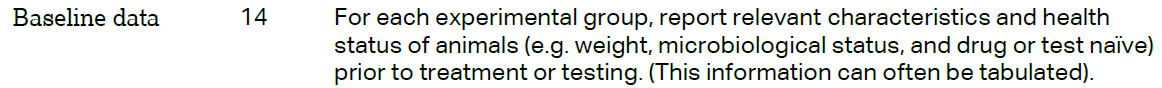 | Line 291-309 | |
| 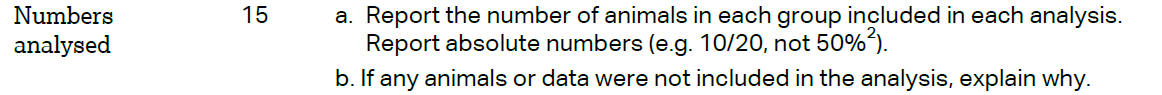 | Line 291-309 | |
| 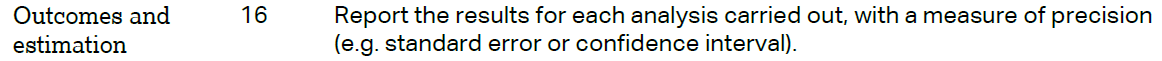 | Figure 5-6 | |
| 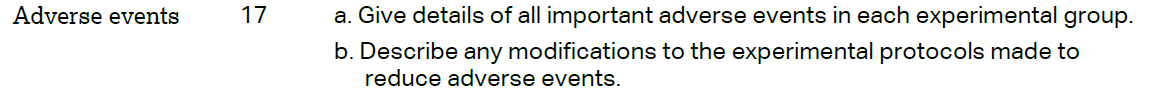 | Not applicable | |
| DISCUSSION |  | |
| 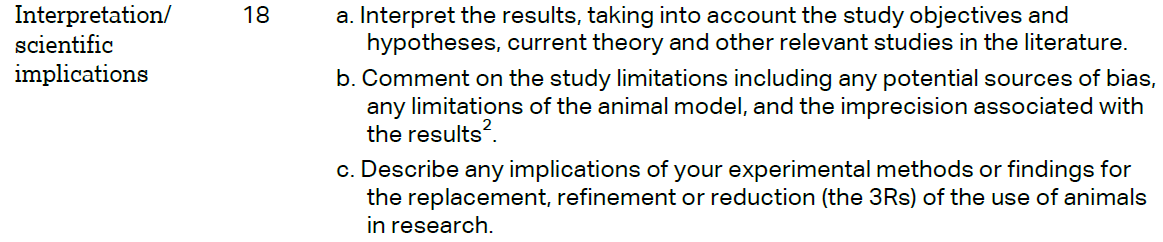 | Discussion: paragraph 1, paragraph 3-4,  paragraph 5-6 | |
| 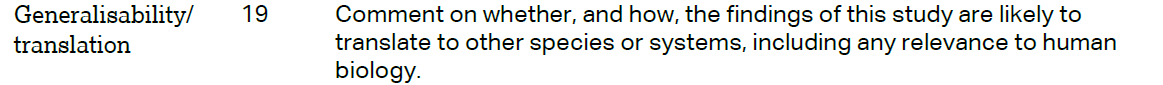 | Discussion: paragraph 5-6 | |
| 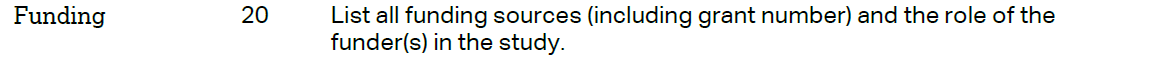 | | Grant Support |


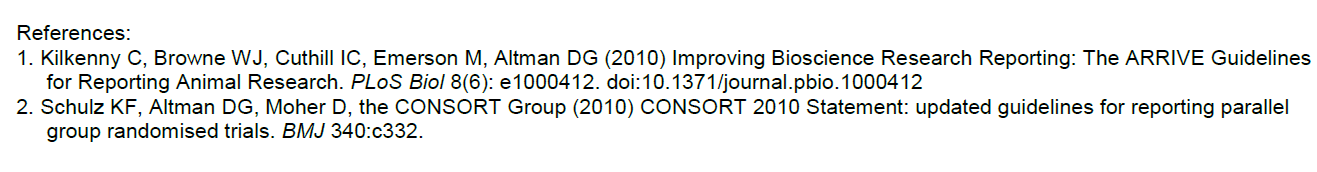

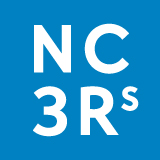

Supplement: S1 Checklist — (DOCX) [file pone.0189044.s005.docx]
